# Sc@B_28_^−^, Ti@B_28_, V@B_28_^+^, and V@B_29_^2−^: Spherically Aromatic Endohedral Seashell-like Metallo-Borospherenes

**DOI:** 10.3390/molecules28093892

**Published:** 2023-05-05

**Authors:** Ting Zhang, Min Zhang, Xiao-Qin Lu, Qiao-Qiao Yan, Xiao-Ni Zhao, Si-Dian Li

**Affiliations:** 1 Institute of Molecular Science, Shanxi University, Taiyuan 030006, China; zhangting0913@sxu.edu.cn (T.Z.); zhangm22@sxu.edu.cn (M.Z.); yanqiaoqiao@sxu.edu.cn (Q.-Q.Y.); zhaoxiaoni@sxu.edu.cn (X.-N.Z.); 2 Department of Chemistry, Xinzhou Teachers’ University, Xinzhou 034000, China; 3 Shanxi Center for Testing of Functional Agro-Products, Shanxi Agricultural University, Taiyuan 030031, China; luxiaoqin@sxu.edu.cn

**Keywords:** first-principles theory, seashell-like metallo-borospherenes, structures, bonding, spherical aromaticity

## Abstract

Transition-metal-doped boron nanoclusters exhibit unique structures and bonding in chemistry. Using the experimentally observed seashell-like borospherenes *C*_2_ B_28_^−/0^ and *C_s_* B_29_^−^ as ligands and based on extensive first-principles theory calculations, we predict herein a series of novel transition-metal-centered endohedral seashell-like metallo-borospherenes *C*_2_ Sc@B_28_^−^ (**1**), *C*_2_ Ti@B_28_ (**2**), *C*_2_ V@B_28_^+^ (**3**), and *C_s_* V@B_29_^2−^ (**4**) which, as the global minima of the complex systems, turn out to be the boron analogues of dibenzenechromium *D_6h_* Cr(C_6_H_6_)_2_ with two B_12_ ligands on the top and bottom interconnected by four or five corner boron atoms on the waist and one transition-metal “pearl” sandwiched at the center in between. Detailed molecular orbital, adaptive natural density partitioning (AdNDP), and iso−chemical shielding surface (ICSS) analyses indicate that, similar to Cr(C_6_H_6_)_2_, these endohedral seashell-like complexes follow the 18-electron rule in bonding patterns (1S^2^1P^6^1D^10^), rendering spherical aromaticity and extra stability to the systems.

## 1. Introduction

Extensive joint photoelectron (PE) spectroscopy and first-principles theory investigations in the past two decades have unveiled a great structural diversity in boron nanoclusters featuring multi-center-two-electron (mc-2e, m ≥ 3) bonding, including the planar or quasi-planar (2D) B_n_^−/0^ (*n* = 3–38, 41, 42) [1,2,3], cage-like *D_2d_* B_40_^−/0^ and *C*_3_/*C*_2_ B_39_^−^ [4,5], and bilayer *D*_2*h*_ B_48_^−/0^[6], with the smallest seashell-like *C*_2_ B_28_^−/0^ [7] and *C_s_* B_29_^−^ [8] observed in gas phases competing with their 2D counterparts in experiments. Based on the experimentally observed cage-like B_40_^−/0^ and B_39_^−^, the borospherene family have been extended to the B*_n_^q^* series (*n* = 36−42, *q* = *n* − 40) in theory [9,10,11]. Theoretical investigations have shown that metal-decorated seashell-like B_28_ may serve as effective potential hydrogen storage materials [12]. The first theoretically predicted perfect cage-like B_80_ in 2007 [13,14] spurred renewed interest in all-boron fullerenes although it was later proved to favor core–shell structures. The bilayer structural motif observed in B_48_^−/0^ has been extended to B_48_-B_72_ and B_84_–B_98_ at the density functional theory (DFT) level, with a bilayer bottom-up approach based on the experimentally observed *C*_6*v*_ B_36_ proposed for the observed bilayer BL-α^+^ borophenes on Ag (111) [15,16,17,18,19]. Mononuclear core–shell B_68_, B_74_, B_80_, B_84,_ B_96_, B_100_, B_101_, B_102,_ and B_112_ and binuclear core–shell *C_s_* B_180_ ((B_12_)_2_@B_156_), *C_s_* B_182_ ((B_12_)_2_@B_158_), and *C_s_* B_184_ ((B_12_)_2_@B_160_) with two interconnected icosahedral B_12_ cores at the center have also been predicted at DFT, with *C_s_* B_112_ and *C_s_* B_184_ as the most stable mononuclear and binuclear species reported to date in thermodynamics [20,21,22,23,24,25,26,27], respectively. Transition-metal doping proves to induce dramatic structural changes in boron nanoclusters. Perfect transition-metal-centered wheel-like *D*_8*h*_ Co©B_8_^−^, *D*_9*h*_ M©B_9_^−^(M = Rh, Ir, Re), and *D*_10*h*_ M©B_10_^−^(M = Ta, Nb), [28,29,30] half-sandwich *C*_3*v*_ CoB_12_^−^ and IrB_12_^−^, and double–ring tubular drum-like *D*_8*d*_ CoB_16_^−^, MnB_16_^−^, and RhB_18_^−^, *C_s_* B_2_–Ta@B_18_^−^, and *D*_10*d*_ Ta@B_20_^−^ have been observed in experiments [31,32,33,34], with Ta@B_20_^−^ possessing the highest coordination number of CN = 20 in tubular species [35]. Perfect lanthanide-metal-doped inverse sandwich *D*_n*h*_ La_2_B_n_^−^ (*n* = 7–9) and spherical trihedral metallo-borospherenes La_3_B_18_^−^ and Tb_3_B_18_^−^ have also been reported in experiments [36,37]. With inspirations from these experimental observations, our group predicted the smallest core–shell spherical trihedral metallo-boronospherene *D_3h_* La_3_[B_2_@B_18_]^−^, perfect spherically aromatic tetrahedral metallo-borospherenes *T_d_* La_4_B_24_ and core–shell *T_d_* La_4_B_29_^0/+/−^ (La_4_[B@B_4_@B_24_]^0/+/−^), endohedral metallo-borospherenes *O_h_* La_6_&[La@B_24_]^+/0^, and the spherically aromatic trihedral metallo-borospherene *D_3h_* La_6_B_30_ in a series of recent papers [38,39,40,41]. Spherical trihedral metallo-borospherenes and endohedral Complexes of B_20_TM_n_ (TM = Sc, Y; *n* = 3, 4) were predicted recently [42]. The Ta-centered metallo-borospherenes Ta@B_22_^−^ and Ta@B_n_^q^ (*n* = 23–28, q = −1–3) which follow the 18-electron rule, the smallest trihedral metallo-borospherene *D*_3*h*_ Ta_3_B_12_^−^ with three equivalent octacoordinate Ta centers in three η^8^–B_8_ rings, and spherical tetrahedral metallo–borospherene *T_d_* Ta_4_B_18_ with four equivalent nonacoordinate Ta centers in four η^9^–B_9_ rings conforming to the 18-electron principle were proposed recently [43,44,45,46]. Alkaline-earth-metal-centered M@B_40_ (M = Ca, Sr) [47] and actinide-metal-centered U@B_40_ [48] were also predicted in theory. However, to the best of our knowledge, spherically aromatic transition-metal-centered endohedral metallo-borospherenes based on the experimentally observed seashell-like *C*_2_ B_28_^−/0^ and *C_s_* B_29_^−^ as the global minima (GM) of the systems have not been reported in the literature.

As boron analogues of benzene (*D*_6*h*_ C_6_H_6_), the experimentally observed quasi-planar *C*_3*v*_ B_12_ with three delocalized π bonds was first utilized as ligands to form the perfect sandwich-like complex *D*_3*d*_ Cr(B_12_)_2_ [49,50]. Unfortunately, such a manually designed complex appears to be a high-lying local minimum of the system unlikely to be produced in experiments. Using the experimentally observed smallest seashell-like borospherenes *C*_2_ B_28_^−/0^ and *C_s_* B_29_^−^ as ligands which contain two B_12_ ligands on the top and bottom interconnected by four or five corner boron atoms on the waist and based on extensive GM searches augmented with first-principles theory calculations, we predict in this work a series of transition-metal-centered seashell-like metallo-borospherenes *C*_2_ Sc@B_28_^−^ (**1**), *C*_2_ Ti@B_28_ (**2**), *C*_2_ V@B_28_^+^ (**3**), and *C_s_* V@B_29_^2−^(**4**) which, as the GMs of the systems with two interconnected B_12_ ligands on the top and bottom and one transition metal center as the “pearl” sandwiched in between, follow the 18-electron rule in bonding patterns, making the transition-metal-doped boron complexes spherically aromatic in nature, highly stable in both thermodynamics and dynamics and possible to be targeted in future experiments.

## 2. Results and Discussions

### 2.1. Structures and Stabilities

**Figure 1:**
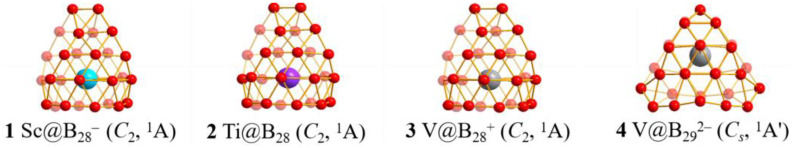
Optimized structures of the transition metal-doped seashell-like endohedral metallo-borospherenes *C*_2_ Sc@B_28_^−^(**1**), *C*_2_ Ti@B_28_ (**2**), *C*_2_ V@B_28_^+^ (**3**), and *C_s_* V@B_29_^2−^ (**4**) at PBE0/6-311+G(d) level.

The obtained transition-metal-centered seashell-like metallo-borospherenes *C_2_* Sc@B_28_^−^ (**1**), *C*_2_ Ti@B_28_ (**2**), *C*_2_ V@B_28_^+^ (**3**), and *C_s_* V@B_29_^2−^ (**4**) as the GMs of the systems at PBE0/6-311+G(d) [51], TPSSh/6-311+G(d) [52,53], and CCSD(T)/6-31G(d) [54,55] levels are collectively depicted in Figure 1, with more alternative low-lying isomers summarized in Figures S1–S4 (ESI†). The isovalent Sc@B_28_^−^ (1), Ti@B_28_ (2), and V@B_28_^+^ (3) with the calculated coordination energies of E_c_ = 9.56, 7.83, 7.57 eV and lowest calculated vibration frequencies of 181.13, 186.63, 184.70 cm^−1^ at PBE0, respectively, turn out to have similar seashell-like structures in the same symmetry as their parent *C*_2_ B_28_ ligand [7], with two B_12_ ligands on the top and bottom interconnected by four corner boron atoms on the waist and one transition metal pearl comfortably sandwiched in between. These axially chiral endohedral metallo-borospherene complexes contain a slightly distorted *C*_2_ B_16_ double-ring tube as the basis of the seashell-like structures, two heptagonal windows on the right and left, and thirty-six B_3_ triangles on the cage surface, with a transition metal center sandwiched comfortably inside the B_28_ cage along the *C*_2_ molecular axis on the upper end of the B_16_ double-ring tube (see detailed coordination bond lengths tabulated in Table S1). *C*_2_ Sc@B_28_^−^ (1), Ti@B_28_ (2), V@B_28_^+^ (3) possess the large calculated HOMO-LUMO energy gaps of Δ*E*_gap_ = 2.10, 2.97, and 3.20 eV at PBE0, respectively, well supporting their high chemical stabilities. It is noticed that the second isomer *C*_2_ Sc&B_28_^−^ (1b) in Figure S1, an exohedral metallo-borospherene with an octacoordinate Sc atom at the lower end of the B_16_ double-ring tube, is actually iso-energetic with Sc@B_28_^−^ (1) at CCSD(T), suggesting that the two degenerate *C*_2_ isomers may coexist in experiments, while, as shown in Figures S2 and S3, the endohedral Ti@B_28_ (2) and V@B_28_^+^ (3) are 0.18 eV and 0.04 eV more stable than their second lowest-lying isomers at CCSD(T), respectively. Triplet and quintet isomers prove to be at least 0.85 eV less stable than their singlet GMs.

The optimized V-centered *C_s_* V@B_29_^2−^ (**4**) also possesses a seashell-like endohedral structure in the same symmetry as its parent ligand *C_s_* B_29_^−^ [8]. It contains two B_12_ ligands on the top and bottom interconnected by five corner boron atoms on the waist, two equivalent octagonal windows on the right and left sides, and thirty-eight B_3_ triangles on the cage surface, with a vanadium center coordinated inside. With a large calculated HOMO-LUOM energy gap of Δ*E*_gap_ = 2.39 eV, coordination energy of E_c_ = 4.79 eV and one small imagery vibrational frequency at −54.30 cm^−1^, *C_s_* V@B_29_^2−^(**4**) appears to be the vibrationally averaged GM of the system between two slightly distorted *C*_1_ V@B_29_^2−^ isomers (4b in Figure S4) in an a″ vibrational mode in which the top B atom and V center swinging left and right reversibly. With zero-point corrections included, *C_s_* V@B_29_^2−^(**4**) turns out to be 0.02 eV and 0.06 eV more stable than the second seashell-like isomer *C*_1_ V@B_29_^2−^ (4b) and third tubular isomer *C_s_* V@B_29_^2−^ (4c) at CCSD(T), respectively (Figure S4). Triplet and quintet isomers are found to be 0.74 eV and 1.81 eV less stable than singlet *C_s_* V@B_29_^2−^(**4**) at PBE0 level, respectively, and all the other isomers lying at least 0.15 eV higher than the *C_s_* GM (**4**).

Detailed natural bonding orbital (NBO) [56] analyses indicate that transition metal centers in Sc@B_28_^−^ (**1**), Ti@B_28_ (**2**), V@B_28_^+^ (**3**), and V@B_29_^2−^ (**4**) possess the net atomic charges 0.76, 0.36, -0.33, and -0.37 |e|, electronic configurations of Sc ([Ar]4s^0.19^3d^1.42^), Ti ([Ar]4s^0.21^3d^2.02^), V ([Ar]4s^0.22^3d^4.26^), and V ([Ar]4s^0.20^3d^4.48^), and total Wiberger bond orders of 4.03, 6.02, 6.70, and 6.44, respectively. Obviously, transition metal coordination centers in these complexes donate their 4s^2^ electrons almost completely to the boron ligands, while in return, accept partial electrons in their partially filled 4d orbitals from the boron ligands via effective π→3d back-donations, enhancing the thermodynamical stabilities of systems.

Extensive Born–Oppenheimer molecular dynamics (BOMD) [57] simulations on Sc@B_28_^−^ (**1**) at 600 K, Ti@B_28_ (**2**) at 700 K, and V@B_29_^2−^ (**4**) at 700 K in Figure S5 clearly indicate that these seashell-like transition metal boron complexes are highly dynamically stable at high temperatures, as evidenced by their small calculated root-mean-square-deviations of RMSD = 0.09, 0.10, 0.10 Å and maximum bond length deviations of MAXD = 0.30, 0.32, 0.33 Å, respectively. No high-lying isomers were observed during the simulations in 30 ps, with the basic structural motifs of the complex systems well maintained in reversible thermal vibrations.

### 2.2. Bonding Pattern Analyses

**Figure 2:**
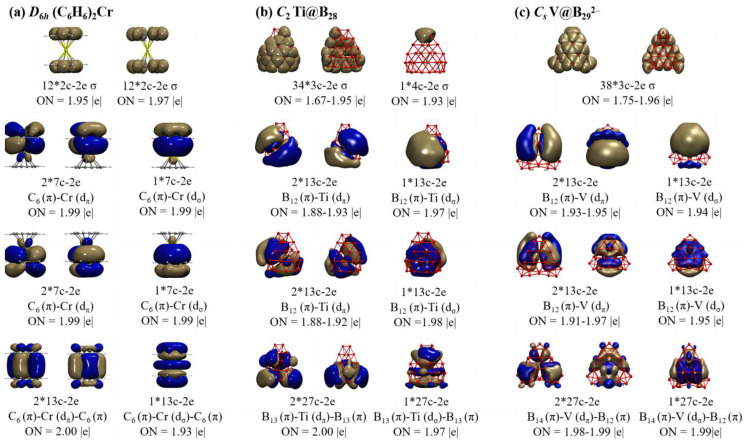
AdNDP bonding patterns of (**a**) *D*_6*h*_ Cr(C_6_H_6_)_2_, (**b**) *C*_2_ Ti@B_28_ (**2**), and (**c**) *C_s_* V@B_29_^2−^ (**4**), with the occupation numbers (ON) indicated.

To better comprehend the high stabilities of these seashell-like endohedral complexes, detailed adaptive natural density partitioning (AdNDP) [58,59] bonding analyses are performed on Ti@B_28_ (**2**) and V@B_29_^2−^ (**4**) in Figure 2, in comparison with that of the prototypic sandwich complex *D*_6*h*_ (C_6_H_6_)_2_Cr. As indicated in Figure 2a, *D*_6*h*_ (C_6_H_6_)_2_Cr possesses 12 2c-2e C-C σ bonds and 12 2c–2e C–H σ bonds on the two C_6_H_6_ ligands with the occupation numbers ON = 1.95 |e|. Its remaining nine delocalized coordination bonds include 3 7c–2e C_6_ (π)–Cr (d_π/σ_) bonds between the Cr center and C_6_H_6_ ligand on the top, 3 7c-2e C_6_ (π)–Cr (d_π/σ_) bonds between the Cr center and C_6_H_6_ ligand at the bottom, and 3 13c C_6_ (π)–Cr (d_π/σ_)–C_6_ (π) bonds between Cr center and the two C_6_H_6_ ligands with ON = 1.93~2.00 |e|, well demonstrating that *D*_6*h*_ (C_6_H_6_)_2_Cr satisfies the 18-electron rule.

Detailed AdNDP analyses presented in Figure 2b indicate that neutral seashell-like *C*_2_ Ti@B_28_ (**2**) contains 34 3c–2e σ bonds on 34 B_3_ triangles on the cage surface and 1 4c–2e σ bond shared by two edge-sharing B_3_ triangles on the upper end, forming the σ-framework of the seashell-like complex. Its remaining nine delocalized coordination bonds include three 13c–2e B_12_ (π)–Ti (d_π/σ_) bonds between the Ti center and B_12_ ligand on the top, three 13c–2e B_12_ (π)–Ti (d_π/σ_) between the Ti center and B_12_ ligand at the bottom, and three 27c–2e B_13_ (π)–Ti (d_π/σ_)–B_13_ (π) bonds mainly between Ti and its two B_12_ ligands on the top and bottom with ON = 1.88~2.00 |e|. Such a delocalized coordination bonding pattern possesses a one-to-one correspondence relationship with that of *D*_6*h*_ (C_6_H_6_)_2_Cr in Figure 2a, indicating that, similar to (C_6_H_6_)_2_Cr, Ti@B_28_ (**2**) follows the 18-electron principle in coordination bonding pattern. Both the isovalent *C*_2_ Sc@B_28_^−^ (**1**) and *C*_2_ V@B_28_^+^ (**3**) are found to follow similar bonding patterns (Figure S6).

*C_s_* V@B_29_^2−^ (**4**) appears to possess a similar bonding pattern. As shown in Figure 2c, it has 38 3c–2e σ bonds on 38 B_3_ triangles on the cage surface, forming the σ-framework of the B_29_^−^ ligand. The remaining nine delocalized coordination bonds include three 13c–2e B_12_ (π)–V(d_π/σ_) bonds between the V center and B_12_ ligand on the top, three 13c–2e B_12_ (π)–V (d_π/σ_) between the V center and B_12_ ligand at the bottom, and three 27c–2e B_14_ (π)–V (d_π/σ_)–B_12_ (π) bonds mainly between V and its two B_12_ ligands on the top and bottom with ON = 1.91~1.99 |e|, again well corresponding to bonding pattern of *D*_6*h*_ (C_6_H_6_)_2_Cr in Figure 2a, showing that V@B_29_^2−^ (**4**) also matches the 18-electron rule in coordination bonding pattern.

The eigenvalue spectra of *D*_6*h*_ (C_6_H_6_)_2_Cr, *C_2_* Ti@B_28_ (**2**), and *C_s_* V@B_29_^2−^ (**4**) compared in Figure S7 indicate that these transition metal-centered complexes possess nine delocalized atomic-like canonical molecular orbitals (CMOs) in the pseudo-superatomic [60] electronic configuration of 1S^2^1P^6^1D^10^ via effective spd-π interaction/hybridizations, indicating that they follow the 18-electron principle and match the 2(n + 1)^2^ electron counting rule (*n* = 2), making them spherically aromatic in nature and chemically stable both thermodynamically and dynamically.

**Figure 3:**
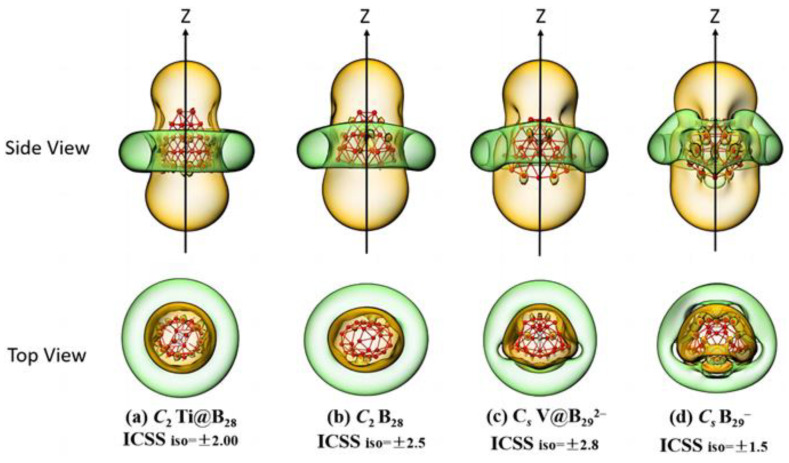
Calculated iso−chemical shielding surfaces (ICSSs) of (**a**) *C*_2_ Ti@B_28_ (**2**) and (**c**) *C_s_* V@B_29_^2−^ (**4**), compared with that of the experimentally known spherically aromatic (**b**) *C*_2_ B_28_ and (**d**) *C_s_* B_29_^−^, respectively.

The calculated iso-chemical shielding surfaces (ICSSs) [61] of Ti@B_28_ (**2**) and V@B_29_^2−^ (**4**) based on the ZZ components of the calculated nuclear-independent chemical shifts (NICS-ZZ) shown in Figure 3a,c appear to be similar with that of the experimentally known spherically aromatic *C*_2_ B_28_ (Figure 3b) [7] and *C_s_* B_29_^−^ (Figure 3d) [8], respectively, well supporting the spherical aromaticity of these endohedral seashell-like endohedral complexes. The spaces inside the boron cage or within 1 Å above the cage surface in vertical directions with negative NICS–ZZ values belong to chemical shielding regions (highlighted in yellow), while the belt-like region outside the cage in the horizontal direction around the waist belongs to the chemical de-shielding area (highlighted in green).

### 2.3. IR, Raman, and PE Spectral Simulations

**Figure 4:**
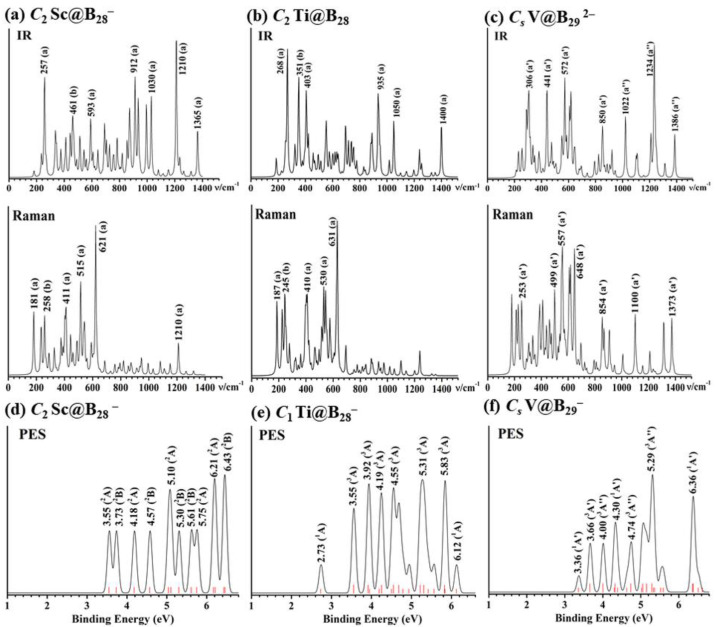
Simulated IR and Raman spectra of (**a**) *C*_2_ Sc@B_28_^−^ (**1**), (**b**) *C*_2_ Ti@B_28_ (**2**), and (**c**) *C_s_* V@B_29_^2−^ (**4**) and PE spectra of (**d**) *C*_2_ Sc@B_28_^−^, (**e**) *C*_1_ Ti@B_28_^−^, and (**f**) *C_s_* V@B_29_^−^ at PBE0/6-311+G(d) level. The red bars in (**d**), (**e**,**f**) stand for the positions of calculated PE features, with the long and short red bars in (**e**,**f**) representing triplet and singlet final states in the neutrals, respectively.

Joint experimental spectroscopic and first-principles theory investigations have proven to be the most effective method to characterize gas phase clusters [62]. The infrared (IR) and Raman spectra of *C*_2_ Sc@B_28_^−^ (**1**), *C*_2_ Ti@B_28_ (**2**), and *C_s_* V@B_29_^2−^ (**3**) are simulated at PBE0/6-311+G(d) in Figure 4 to facilitate their future spectroscopic characterizations. As shown in Figure 4a, *C*_2_ Sc@B_28_^−^ (**1**) exhibits strong IR active peaks at 257 (a), 461 (b), 593 (a), 872 (a), 912 (a), 936 (b), 1030 (a), 1210 (a), and 1365 (a) cm^−1^ which mainly belong to the vibrational modes of the B_28_ skeleton, while its strong Raman active vibrations occur at 181 (a), 258 (b), 411 (a), 515 (a), 621 (a), 1210 cm^−1^ (a), with the 411 cm^−1^ (a) peak corresponding to typical “radial breathing mode” (RBM) [63] of the *C_2_* B_28_ ligand which can be used to characterize hollow boron nanostructures. The IR and Raman spectra of Ti@B_28_ (Figure 4b) is similar to that of Sc@B_28_^−^, with the IR active vibrational modes at 268 (a), 351 (b), 403 (a), 935 (a), 1050 (a), and 1400 (a) and Raman active vibrations at 187 (a), 245 (b), 410 (a), 530 (a), and 631(a) cm^−1^, respectively, with the 530 cm^−1^ (a) peak belonging to typical RBM. The strong IR peaks of V@B_29_^2−^ (**4**) occur at 306 (a′), 441 (a′), 572 (a′), 850 (a′), 1022 (a″), 1234 (a″), and 1386 (a″), while its Raman features are located at 253 (a′), 499 (a′), 557 (a′), 648 (a′), 854 (a″), 1100 (a′), and 1373 (a″) (Figure 4c). Simulated IR and Raman spectra of (a) *C*_2_ V@B_28_^+^ are shown in Figure S8.

The simulated PE spectra of *C*_2_ Sc@B_28_^−^ (**1**) and *C*_1_ Ti@B_28_^−^ and *C_s_* V@B_29_ ^−^ derived from *C_2_* Ti@B_28_ (**2**) and *C_s_* V@B_29_^2−^ (**4**) are shown in Figure 4d–f using the time-dependent TD-PBE0/6-311+G(d) approach [64,65], with their first calculated vertical detachment energies (VDEs) located at 3.55, 2.73, and 3.36 eV and first adiabatic detachment energies (ADEs) located at 3.33, 2.41, and 3.21 eV, respectively. Detachment of one electron from singlet *C*_2_ Sc@B_28_^−^ (**1**) leads to doublet final states in its neutral, with the major spectroscopic features at 3.55, 3.73, 4.18, 4.57, 5.10, 5.30, 5.61, 5.75, 6.21, and 6.43 eV, respectively (Figure 4d). Detachment of one electron from the open-shell doublet *C*_1_ Ti@B_28_^−^ and *C_s_* V@B_29_^−^ generates both singlet or triplet final states in their neutrals, with the major spectral peaks located at 2.73, 3.55, 3.92, 4.19, 4.55, 5.31, 5.83, and 6.12 eV for Ti@B_28_^−^ and 3.36 3.66, 4.00, 4.30, 4.74, 5.29, and 6.36 eV for V@B_29_^−^, respectively (Figure 4e,f).

## 3. Computational Details

Extensive GM searches were performed on Sc@B_28_^−^, Ti@B_28_, and V@B_28_^+^, V@B_29_^2−^ at DFT level with electronic multiplicities considered, using both the TGmin2 [66,67] and Minima Hopping (MH) [68,69] codes, in conjunction with manual constructions based on the experimentally observed *C_2_* B_28_^−/0^ and *C_s_* B_29_^−^ at PBE/DZVP, with about 3500 stationary points probed for each species on its potential surface. The low-lying isomers were then fully optimized at both PBE0/6-311+G(d) [51] and TPSSh/6-311+G(d) [52,53] levels using the Gaussian 09 program, with vibrational frequencies checked to make sure all the obtained low-lying isomers are true minima of the systems. Single point CCSD(T)/6-31G(d) calculations were performed on the five lowest–lying isomers to further refine their relative energies employing the Molpro (2013) program [54,55], with the T_1_ diagnostics checked to make sure that multi-reference interactions make non-significant contributions in these closed-shell complexes. Natural bonding orbital (NBO) analyses were carried out using the NBO 6.0 program [56]. Extensive Born–Oppenheimer molecular dynamics (BOMD) simulations were performed on *C*_2_ Sc@B_28_^−^(**1**) at 600 K, *C*_2_ Ti@B_28_ (**2**) at 700 K, and V@B_29_^2−^(**4**) at 700 K for 30 ps using the CP2K program [57] utilizing the hybrid Gaussian and plane waves method, with the GTH–PBE pseudopotential and DZVP–MOLOPT–SR–GTH basis set for boron and transition metal, respectively. Detailed bonding analyses were carried out utilizing the adaptive natural density partitioning (AdNDP) approach [58,59]. Iso-chemical shielding surfaces (ICSS) [61] were calculated using the Multiwfn 3.8 software [70]. Bonding analyses and ICSS surfaces were visualized using the visual molecular dynamics (VMD) [71] software. The IR and Raman spectra of *C*_2_ Sc@B_28_^−^ (**1**), *C*_2_ Ti@B_28_ (**2**), *C_s_* V@B_29_^2−^ (**4**) were simulated at PBE0/6-311+G(d). The PE spectra of *C*_2_ Sc@B_28_^−^ (**1**), *C*_1_ Ti@B_28_^−^ and *C_s_* V@B_29_^−^ were simulated using the time-dependent DFT approach (TD-DFT) at PBE0/6-311+G(d) level [64,65]. An overall calculation scheme used in this work is presented in Figure S9.

## 4. Conclusions

Based on the experimentally observed seashell-like *C_2_* B_28_^−/0^ and *C_s_* B_29_^−^ and extensive first-principles theory calculations, we propose in this work the transition-metal-centered endohedral seashell-like metallo-borospherenes Sc@B_28_^−^ (**1**), Ti@B_28_ (**2**), V@B_28_^+^ (**3**), and V@B_29_^2−^ (**4**) which, as the boron analogues to the well-known sandwich complex Cr(C_2_H_6_)_2_ highly stable both thermodynamically and dynamically, follow the 18-electron rule in coordination bonding patterns and are spherically aromatic in nature. The IR, Raman, and PE spectra of the concerned species are theoretically simulated to facilitate their future spectroscopic characterizations in gas-phase experiments via laser ablations of boron-transition-metal mixed binary targets. Further combined theoretical and experimental investigations on metal-doped boron complexes are expected to unveil novel structures and bonding in chemistry and materials science and shed new insights on boron-based nano-devices.

## Data Availability

Not applicable.

## Supplementary Materials

The following supporting information can be downloaded at: https://www.mdpi.com/article/10.3390/molecules28093892/s1, Figure S1: Low-lying isomers of *C*_2_ Sc@B_28_^−^ with their relative energies; Figure S2: Low-lying isomers of *C*_2_ Ti@B_28_ with their relative energies; Figure S3: Low-lying isomers of *C*_2_ V@B_28_^+^ with their relative energies; Figure S4: Low-lying isomers of *C_s_* V@B_29_^2−^ with their relative energies; Figure S5: Molecular dynamics simulations of (a) Sc@B_28_^−^ (1) at 600 K, (b) Ti@B_28_ (2) at 700 K, and (c) V@B_29_^2−^ (4) at 700 K; Figure S6: AdNDP Analysis of (a) *C*_2_ Sc@B_28_^−^ and (b) *C*_2_ V@B_28_^+^; Figure S7: Molecular orbital energy levels of (a) D*_6h_* (C_6_ H_6_)_2_Cr, (b) *C*_2_ Ti@B_28_ and (c) *C_s_* V@B_29_^2−^; Figure S8: Simulated IR and Raman spectra of (a) *C_2_* V@B_28_^+^; Figure S9: An overall scheme of the theoretical procedures adapted in this work. Table S1: The bond lengths r_Sc-B_ of *C*_2_ Sc@B_28_^−^, r_Ti-B_ of *C*_2_ Ti@B_28_, r_V-B_ of *C*_2_ V@B_28_ and r’_V-B_ of *C_s_* V@B_29_^2−^; Table S2: Cartesian coordinates of the optimized low-lying isomers.

## Sample Availability

Samples of the compounds are not available from the authors.
