# Supplementary material for: Sc@B28−, Ti@B28, V@B28+, and V@B292−: Spherically Aromatic Endohedral Seashell-like Metallo-Borospherenes
Source: Molecules. 2023 May 5;28(9):3892. doi: 10.3390/molecules28093892 (PMC10179789; doi:10.3390/molecules28093892)
Supplement: Supplementary file 1 [file molecules-28-03892-s001.zip › molecules-2343164-supplementary.pdf]

SUPPLEMENTARY INFORMATION

$Sc@B_{28}^{-}$, $Ti@B_{28}$, $V@B_{28}^{+}$, and $V@B_{29}^{2-}$: Spherically Aromatic Endohedral Seashell-Like Metallo-Borospherenes

Ting Zhang [1,2], Min Zhang [1], Xiao-Qin Lu [3], Qiao-Qiao Yan [1], Xiao-Ni Zhao [1] and Si-Dian Li [1,*]

[1] Institute of Molecular Science, Institute of Molecular Science, Shanxi University, Taiyuan 030006, China; zhangting0913@sxu.edu.cn (T.Z.); zhangm22@sxu.edu.cn (M.Z.); yanqiaoqiao@sxu.edu.cn (Q.-Q.Y.); zhaoxiaoni@sxu.edu.cn (X.-N.Z.)

[2] Department of Chemistry, Xinzhou Teachers' University, Xinzhou 034000, China

[3] Shanxi Center for Testing of Functional Agro-Products, Shanxi Agricultural University, Taiyuan 030031, China; luxiaoqin@sxu.edu.cn

* Correspondence: lisidian@sxu.edu.cn

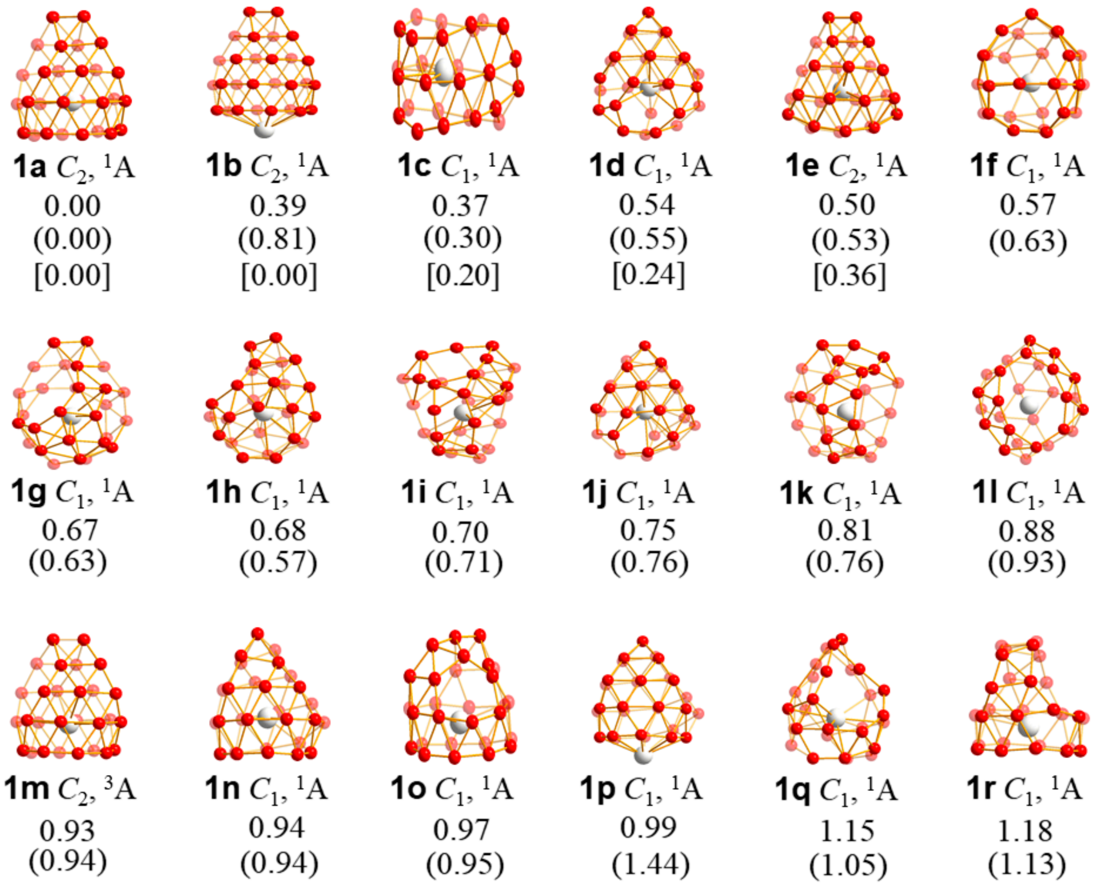

Figure S1. Low-lying isomers of C_2 Sc@B_{28}^- with their relative energies indicated in eV at PBE0/6-311+G(d), TPSSh/6-311+G(d) (parentheses), and CCSD(T)/6-31G(d) (square brackets) levels, respectively.

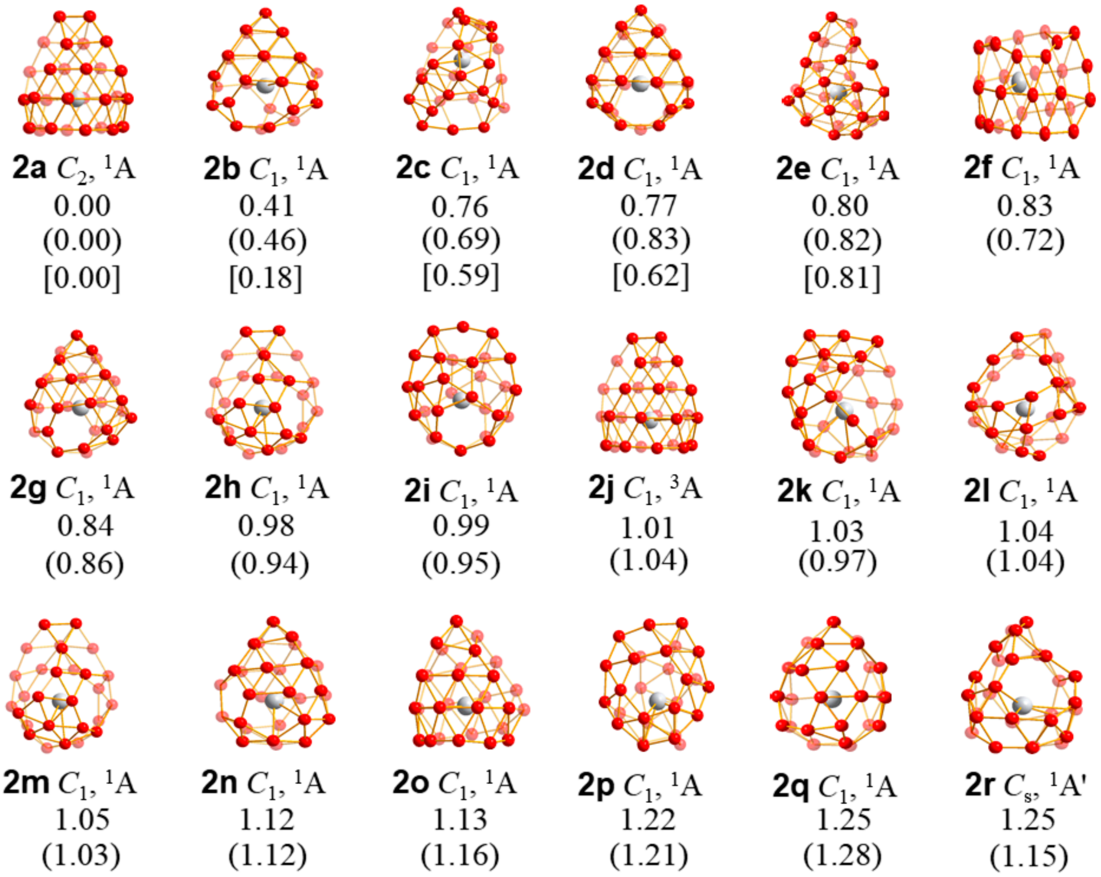

Figure S2. Low-lying isomers of C_2 Ti@B_{28} with their relative energies indicated in eV at PBE0/6-311+G(d), TPSSh/6-311+G(d) (parentheses), and CCSD(T)/6-31G(d) (square brackets) levels, respectively.

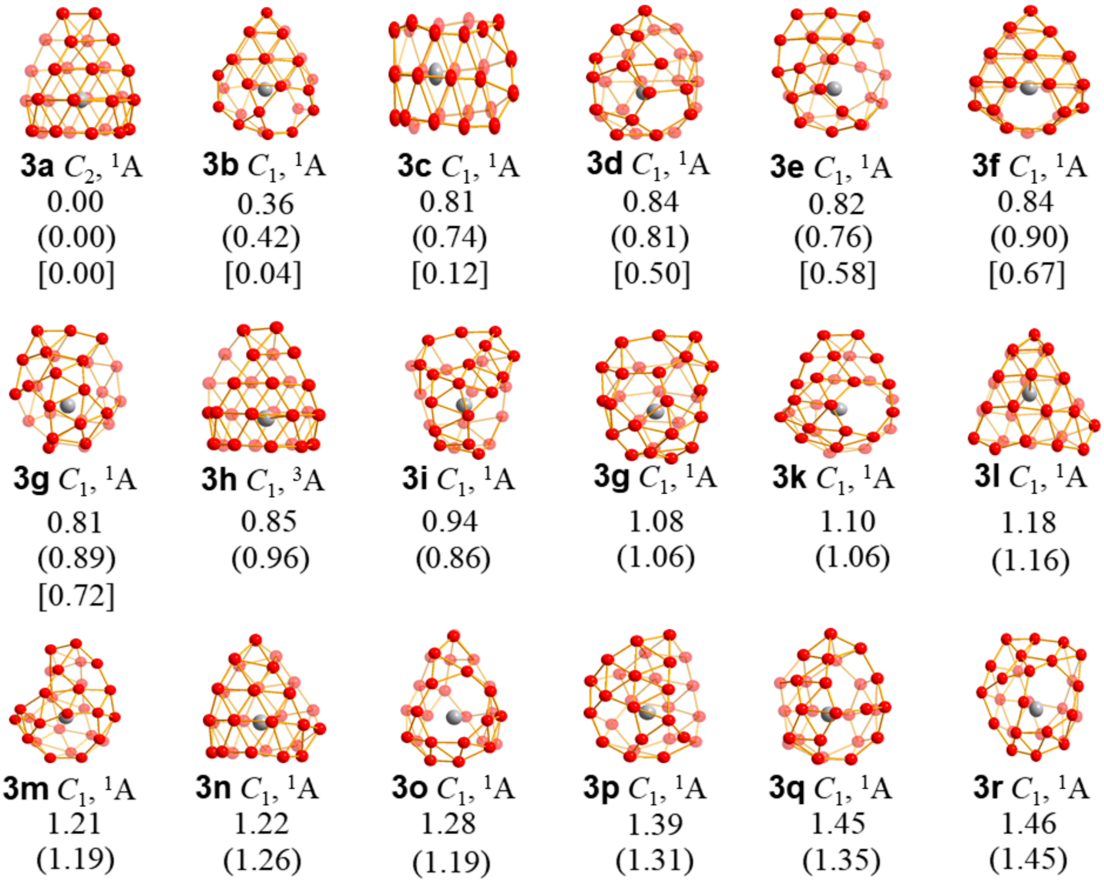

Figure S3. Low-lying isomers of C_2 V@B_{28}^+ with their relative energies indicated in eV at PBE0/6-311+G(d), TPSSh/6-311+G(d) (parentheses), and CCSD(T)/6-31G(d) (square brackets) levels, respectively.

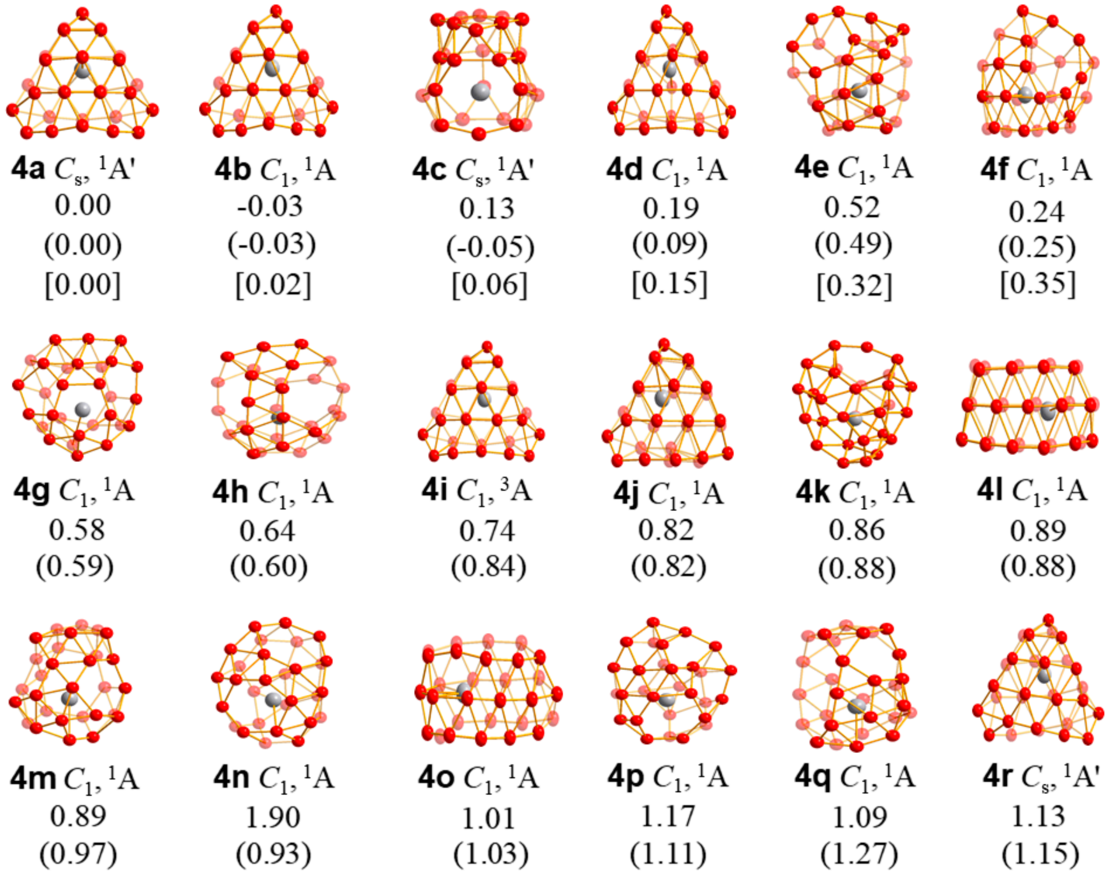

Figure S4. Low-lying isomers of C_s $V@B_{29}^{2-}$ with their relative energies indicated in eV at PBE0/6-311+G(d), TPSSh/6-311+G(d) (parentheses), and CCSD(T)/6-31G(d) (square brackets) levels, respectively.

Table S1. The bond lengths $r_{Sc\text{-}B}$ of C_2 Sc@B_{28}^{-}, $r_{Ti\text{-}B}$ of C_2 Ti@B_{28}, $r_{V\text{-}B}$ of C_2 V@B_{28} and $r'_{V\text{-}B}$ of C_s V@B_{29}^{2-}.

Atomic labels*	Sc@B_{28}^{-} $r_{Sc\text{-}B}$ (Å)	Ti@B_{28} $r_{Ti\text{-}B}$ (Å)	V@B_{28}^{+} $r_{V\text{-}B}$ (Å)	V@B_{29}^{2-} $r'_{V\text{-}B}$ (Å)
1	2.7825	2.8372	2.9053	2.2205
2	2.3148	2.2642	2.2232	2.5322
3	2.5772	2.5393	2.5177	2.3505
4	2.3775	2.3492	2.3337	2.2199
5	2.5830	2.5705	2.5617	2.4499
6	2.5480	2.5219	2.4971	2.3505
7	2.3962	2.3664	2.3487	2.3718
8	3.1862	3.2512	3.3023	2.5322
9	2.6476	2.6402	2.6389	2.3718
10	2.6476	2.3664	2.6389	2.6266
11	2.3962	2.3664	2.3487	2.8383
12	2.3683	2.3153	2.2726	2.6438
13	2.5302	2.5050	2.4971	3.5503
14	2.4515	2.4273	2.4116	3.8476
15	2.4515	2.4273	2.4116	3.5503
16	2.3148	2.2642	2.2232	3.6991
17	2.5772	2.5050	2.5177	2.2913
18	2.3775	2.3492	2.3337	3.6991
19	2.3683	2.3153	2.2726	2.6438
20	4.0566	4.0782	4.1035	3.5109
21	2.5302	2.5050	2.4971	2.4123
22	2.8132	2.7942	2.7867	2.4123
23	2.5480	2.5219	2.4971	2.8383
24	2.7825	2.8372	2.9053	2.1935
25	3.1862	3.2512	3.3023	2.2913
26	2.8132	2.7942	2.7867	2.4303
27	2.5830	2.5705	2.5617	2.1999
28	4.0566	4.0782	4.1035	2.6266
29	---	---	---	3.8476

*** Atomic labels used in Table S1**

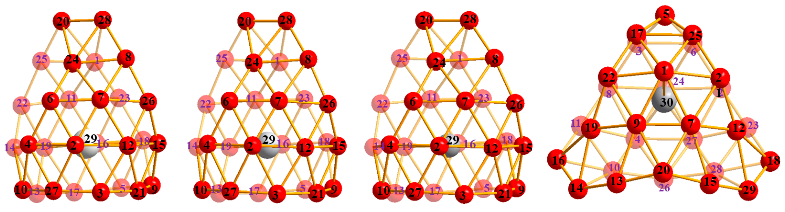

C_2 Sc@B_{28}^{-} C_2 Ti@B_{28} C_2 V@B_{28} C_s V@B_{29}^{2-}

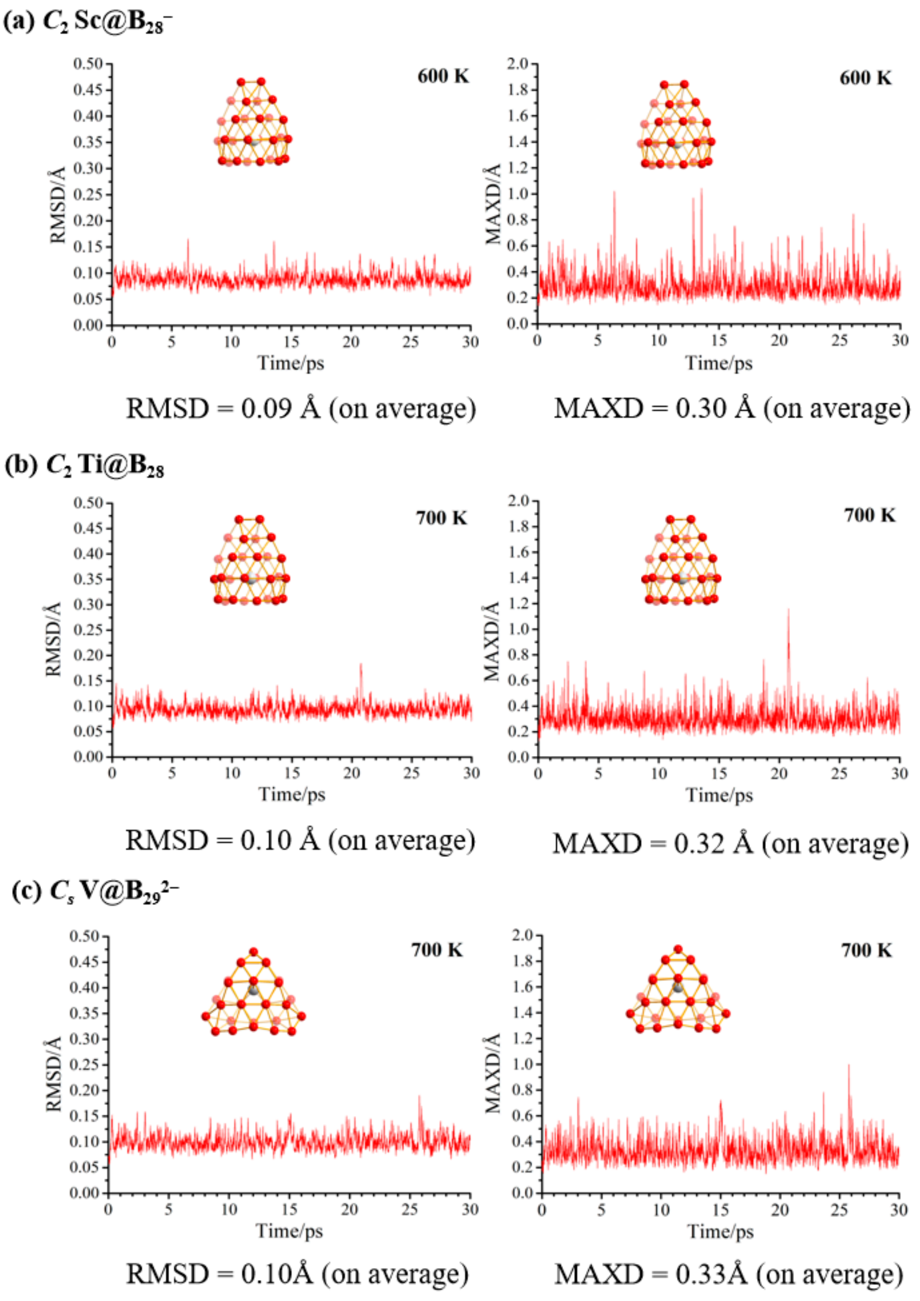

Figure S5. Molecular dynamics simulations of (a) Sc@B_{28}^- (**1**) at 600 K, (b) Ti@B_{28} (**2**) at 700 K, and (c) V@B_{29}^{2-} (**4**) at 700 K, respectively, with the average root-mean-square-deviations (RMSD) and maximum bond length deviations (MAXD) indicated in Å.

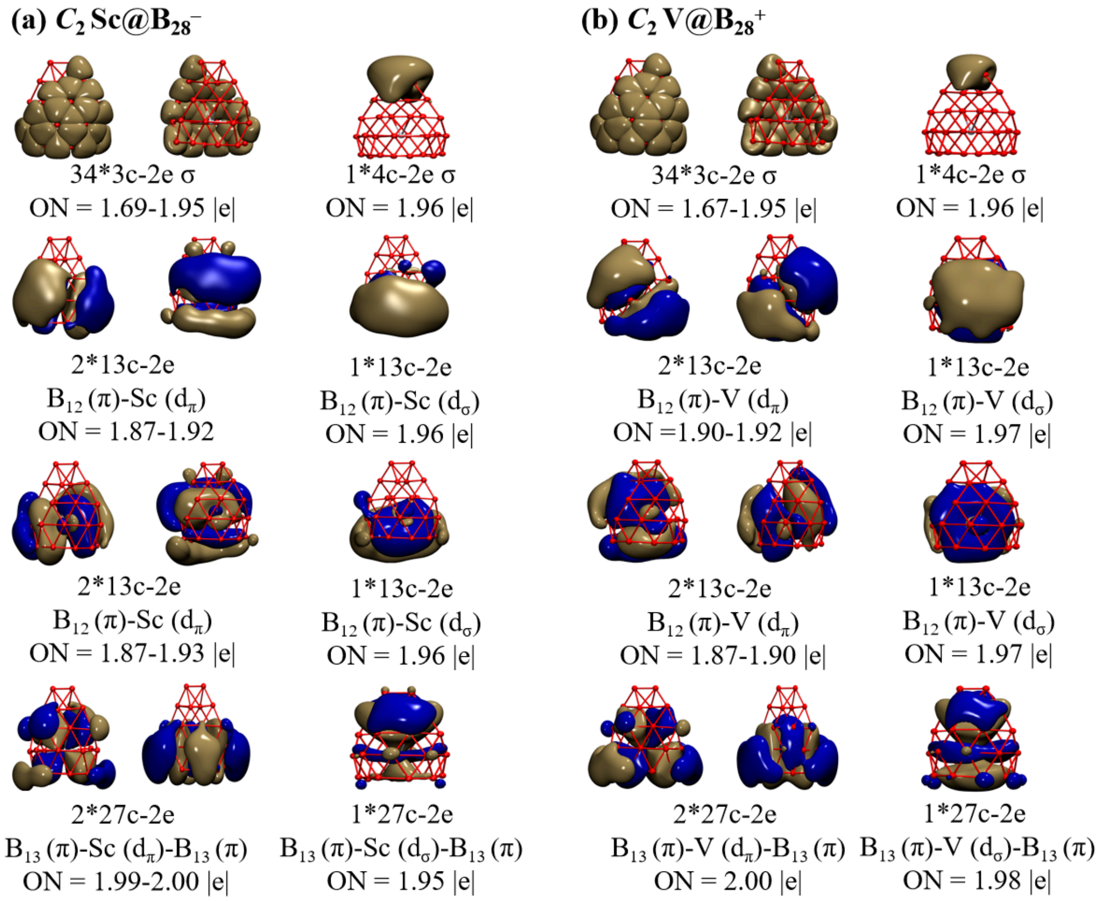

Figure S6. AdNDP Analysis of (a) C_2 Sc@B_{28}^{-} and (b) C_2 V@B_{28}^{+} .

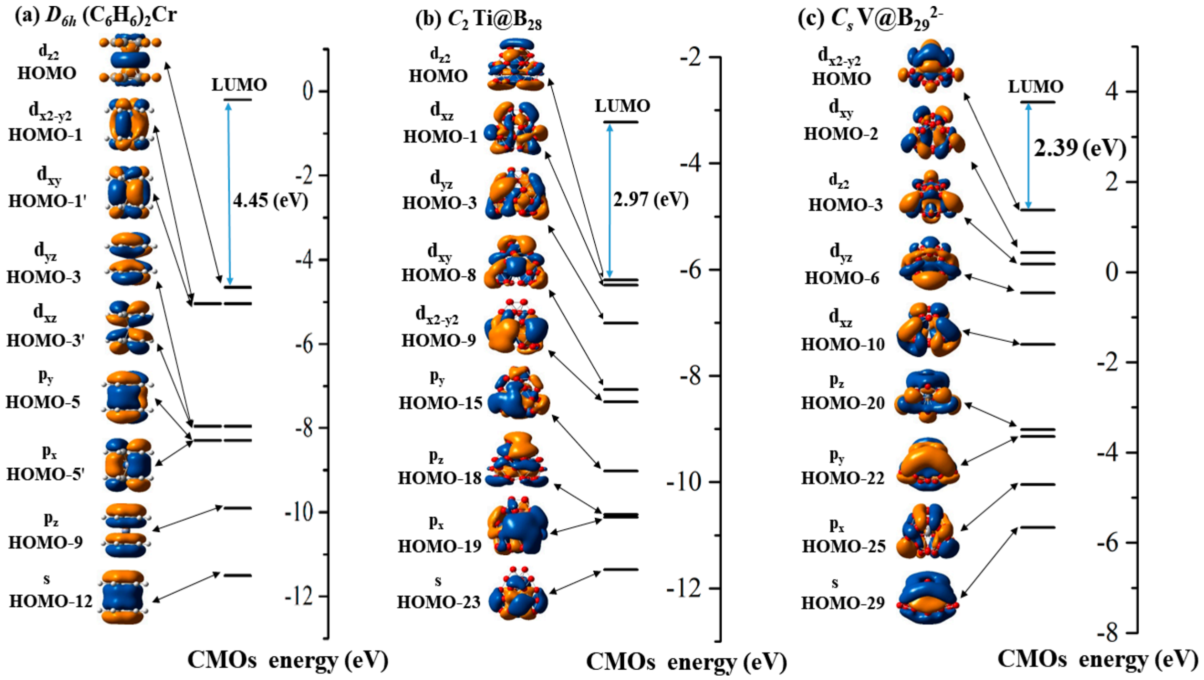

Figure S7. Molecular orbital energy levels of (a) D_{6h} $(C_6H_6)_2Cr$, (b) C_2 Ti@B_{28} and (c) C_s V@B_{29}^{2-} at PBE0/6 311+G(d) level.

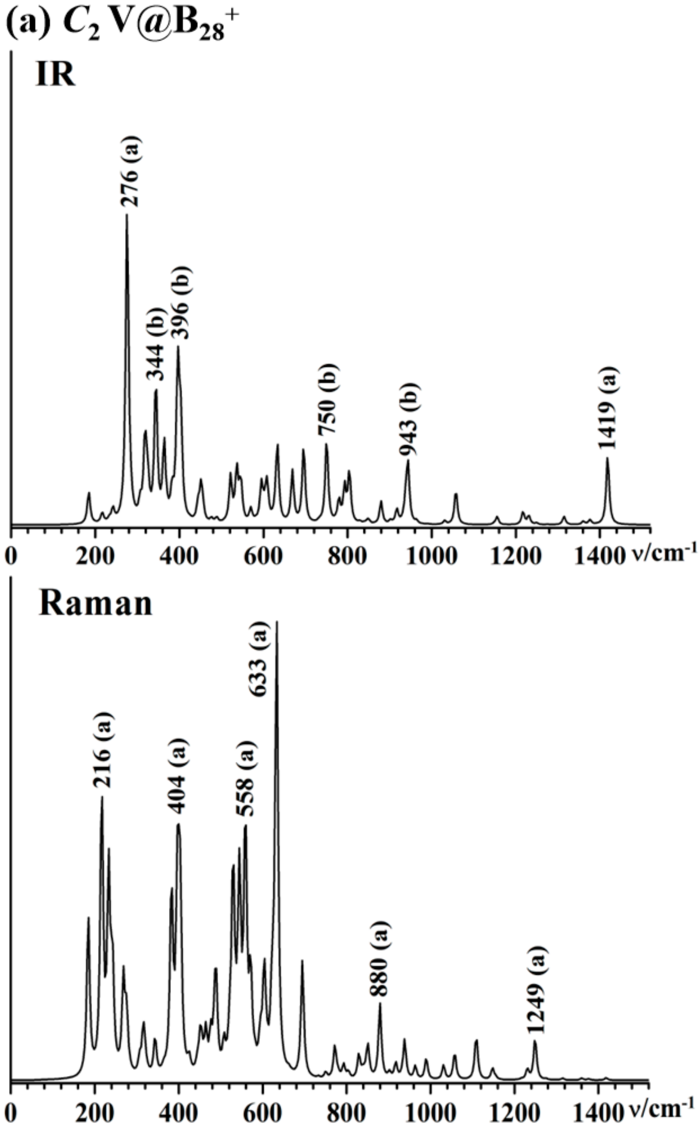

Figure S8. Simulated IR and Raman spectra of (a) C_2 V@B$_{28}$$^+$ at PBE0/6-311+G(d) level.

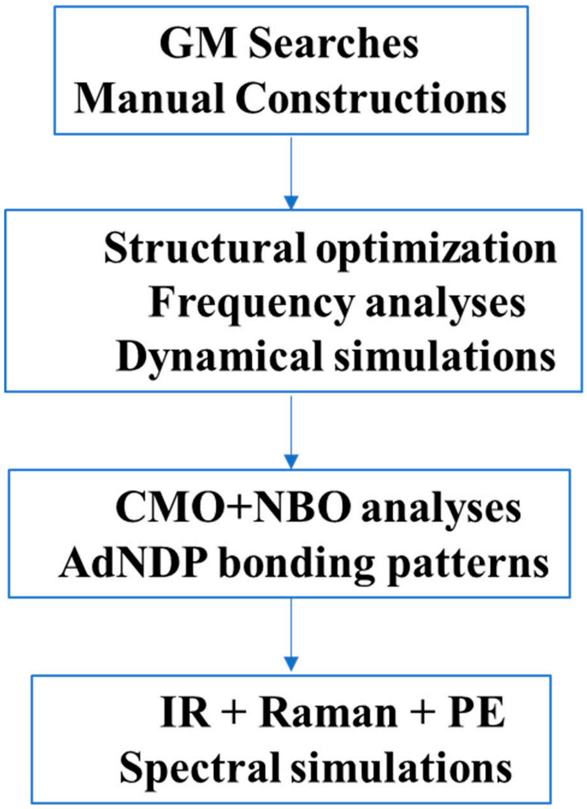

Figure S9. An overall scheme of the theoretical procedures adapted in this work.

Table S2. Cartesian coordinates of the optimized low-lying isomers.

(1a) $Sc@B_{28}^{-}$ (C_2, ^1A)

B	-0.48777100	-0.81143100	-2.16047000
B	1.64953300	1.62399500	0.46670000
B	1.88600300	0.79896000	2.02013700
B	0.00000000	2.37707800	0.40994300
B	-0.78790800	-1.97485100	1.92262600
B	0.76281000	1.96588700	-0.97418600
B	1.81144400	0.65293600	-0.97023200
B	1.54395600	-0.65122500	-2.25391700
B	0.82971200	-2.08037800	1.86791500
B	-0.82971200	2.08037800	1.86791500
B	-1.81144400	-0.65293600	-0.97023200
B	2.36627300	-0.03264500	0.54714700
B	-1.82082300	0.80231500	2.01900600
B	-1.75711900	1.70855100	0.51097300
B	1.75711900	-1.70855100	0.51097300
B	-1.64953300	-1.62399500	0.46670000
B	-1.88600300	-0.79896000	2.02013700
B	0.00000000	-2.37707800	0.40994300
B	-2.36627300	0.03264500	0.54714700
B	-0.68205800	0.36408200	-3.52621700
B	1.82082300	-0.80231500	2.01900600
B	-2.29843200	0.97934500	-0.83705400
B	-0.76281000	-1.96588700	-0.97418600
B	0.48777100	0.81143100	-2.16047000
B	-1.54395600	0.65122500	-2.25391700
B	2.29843200	-0.97934500	-0.83705400
B	0.78790800	1.97485100	1.92262600
B	0.68205800	-0.36408200	-3.52621700
Sc	0.00000000	0.00000000	0.45601500

(1b) $Sc@B_{28}^{-}$ (C_1, ^1A)

B	0.45533200	0.77496100	-2.52505700
B	-1.53867000	-1.46288900	0.10972100
B	-1.96899700	-0.74651500	1.61680800
B	0.00000000	-2.27144500	0.10295500
B	0.81708400	1.87963900	1.58544900
B	-0.74115500	-1.89667600	-1.29262000
B	-1.65445000	-0.51018900	-1.28112300
B	-1.43238000	0.72534000	-2.57161700
B	-0.79213100	2.06515300	1.59803600
B	0.79213100	-2.06515300	1.59803600
B	1.65445000	0.51018900	-1.28112300
B	-2.31108400	0.14215000	0.18105400
B	1.86174800	-0.86103600	1.67586500
B	1.67910100	-1.74220500	0.19464700
B	-1.67910100	1.74220500	0.19464700
B	1.53867000	1.46288900	0.10972100
B	1.96899700	0.74651500	1.61680800
B	0.00000000	2.27144500	0.10295500
B	2.31108400	-0.14215000	0.18105400
B	0.69378400	-0.34477500	-3.92773500
B	-1.86174800	0.86103600	1.67586500
B	2.27039400	-1.06837000	-1.17559700
B	0.74115500	1.89667600	-1.29262000
B	-0.45533200	-0.77496100	-2.52505700
B	1.43238000	-0.72534000	-2.57161700
B	-2.27039400	1.06837000	-1.17559700
B	-0.81708400	-1.87963900	1.58544900
B	-0.69378400	0.34477500	-3.92773500
Sc	0.00000000	0.00000000	2.71867300

(1c) Sc@B_{28}^{-} (C_1, ^1A)

B	1.29928800	-2.13189700	0.57238700
B	1.41300000	2.10469400	-0.55890100
B	-3.20492000	-0.50194000	-0.39997400
B	2.62835600	-0.87550500	0.22806000
B	2.63940700	0.80187200	-0.23775100
B	-0.34938500	2.24789900	-0.42969400
B	-1.90028800	1.62820800	-0.16455700
B	-1.88179500	-1.53576800	0.17734600
B	-0.38446700	-2.32786000	0.56334100
B	0.46588100	-2.49826000	-0.88869800
B	1.70055300	0.80751700	-1.97389700
B	-1.57912600	-0.25040700	-1.66290100
B	1.96864700	-1.86130200	-0.95376300
B	2.54462200	-0.41715200	-1.43016400
B	0.44782900	1.72605600	-1.89938900
B	-1.14999900	1.33085500	-1.63382900
B	-2.57492900	-1.57852400	-1.37513700
B	-0.96990800	-1.85419700	-1.39723800
B	0.40439400	-1.63197000	1.97846100
B	2.10552000	1.83410700	0.95197100
B	1.69926300	-0.80169800	2.06316800
B	2.58531600	0.32331600	1.40410200
B	-2.20251800	-0.17918400	1.23104500
B	-1.18674400	-1.34777500	1.70014600
Sc	0.36770700	-0.01699900	-0.00998600
B	-3.35500500	0.87584300	0.44184900
B	0.61697300	2.45597500	0.99855800
B	-0.92032200	2.17381100	1.25912100
B	-2.40401000	1.55468200	1.47827700

(1d) Sc@B_{28}^{-} (C_1, ^1A)

B	1.23499100	0.40199700	-1.74811600
B	-0.17794500	0.23367800	2.50143300
B	1.07337000	-1.97179000	0.73991900
B	-0.23654600	1.31088200	-1.95370300
B	0.16663900	-2.23853000	-0.86179600
B	1.12827900	1.94052900	-0.85463800
B	1.22315400	-1.32430700	-1.76740000
B	-0.43042600	-2.51600600	0.73092700
B	2.45672300	1.04119400	-0.72353300
B	-2.70333400	0.41429000	-0.68087700
B	-1.74070000	1.67451400	-1.10572900
B	-0.11147400	-0.33741900	-2.40043600
B	0.30169300	2.32099300	0.77120400
B	-2.56427100	-1.19249200	-0.63052800
B	2.42046400	-1.04450600	0.75155300
B	-1.62855400	-0.26896400	2.03998700
B	-1.27307100	-1.34943900	-1.77835700
B	-0.27621000	-1.36699700	1.92740000
B	2.58879800	-0.60322600	-1.10683500
B	-2.75664800	-0.35732100	0.81464600
B	3.51576600	0.04285500	0.09757900
B	-2.31264000	1.17371000	0.96250400
B	1.05575700	1.28446700	1.83802900
B	-1.34085400	2.37876500	0.54945900
B	-1.45682600	-2.38619700	-0.53281400
B	-0.39344200	2.49936600	-0.76946800
B	2.49288000	0.55920800	1.27283600
B	1.19694300	-0.42496500	1.81463200
Sc	-0.34583700	0.02516900	0.02431400

(1e) Sc@B_{28}^{-} (C_1, ^{1}A)

B	-0.60740800	-1.01887600	-2.13237500
B	1.43699600	1.79161600	0.61632700
B	1.37816400	0.79188100	2.16588500
B	0.00000000	2.63848100	0.42386600
B	-0.12529800	-1.77845100	1.91937700
B	0.71407200	2.17547800	-0.96285900
B	1.87970800	0.96345400	-0.90326600
B	1.80153000	-0.30233900	-2.11263500
B	1.32049900	-2.31370600	1.34101800
B	-1.32049900	2.31370600	1.34101800
B	-1.87970800	-0.96345400	-0.90326600
B	2.31336900	0.31250100	0.72193000
B	-1.54656000	0.79173500	1.97851200
B	-2.40977600	1.38408400	0.58671100
B	2.40977600	-1.38408400	0.58671100
B	-1.43699600	-1.79161600	0.61632700
B	-1.37816400	-0.79188100	2.16588500
B	0.00000000	-2.63848100	0.42386600
B	-2.31336900	-0.31250100	0.72193000
B	-0.68742300	0.33297600	-3.27832800
B	1.54656000	-0.79173500	1.97851200
B	-2.64306900	0.53766200	-0.75258300
B	-0.71407200	-2.17547800	-0.96285900
B	0.60740800	1.01887600	-2.13237500
B	-1.80153000	0.30233900	-2.11263500
B	2.64306900	-0.53766200	-0.75258300
B	0.12529800	1.77845100	1.91937700
B	0.68742300	-0.33297600	-3.27832800
Sc	0.00000000	0.00000000	0.18496200

(2a) Ti@B_{28} (C_2, ^1A)

B	-0.50142100	-0.82331300	-2.19343600
B	1.62168200	1.58009500	0.46172900
B	1.85537900	0.77583900	2.02533600
B	0.00000000	2.34823200	0.40808700
B	-0.79703800	-1.97037000	1.92061500
B	0.72691700	1.92892800	-0.97795900
B	1.76768400	0.61950900	-0.97129100
B	1.56504200	-0.67570400	-2.29356500
B	0.80786700	-2.08168400	1.88370900
B	-0.80786700	2.08168400	1.88370900
B	-1.76768400	-0.61950900	-0.97129100
B	2.31362600	-0.05671900	0.54113400
B	-1.79684000	0.81588100	2.01794700
B	-1.71709900	1.71517800	0.51233900
B	1.71709900	-1.71517800	0.51233900
B	-1.62168200	-1.58009500	0.46172900
B	-1.85537900	-0.77583900	2.02533600
B	0.00000000	-2.34823200	0.40808700
B	-2.31362600	0.05671900	0.54113400
B	-0.67359200	0.38011400	-3.52925300
B	1.79684000	-0.81588100	2.01794700
B	-2.23999500	1.01694100	-0.85022800
B	-0.72691700	-1.92892800	-0.97795900
B	0.50142100	0.82331300	-2.19343600
B	-1.56504200	0.67570400	-2.29356500
B	2.23999500	-1.01694100	-0.85022800
B	0.79703800	1.97037000	1.92061500
B	0.67359200	-0.38011400	-3.52925300
Ti	0.00000000	0.00000000	0.47492500

(2b) Ti@B_{28} (C_1, ^1A)

B	1.22426800	0.21800600	-1.68563200
B	-0.15636700	0.47481700	2.42014200
B	1.04903800	-1.84676600	0.82514700
B	-0.19489300	1.12316400	-2.02583500
B	0.13459000	-2.29302300	-0.67192500
B	1.13771100	1.80105900	-0.93906700
B	1.23398500	-1.48998900	-1.61362200
B	-0.44759500	-2.42009400	0.94001900
B	2.49763500	0.93526100	-0.76311200
B	-2.66199400	0.35136400	-0.72273600
B	-1.71077900	1.56144900	-1.24777900
B	-0.11249700	-0.54712100	-2.31139400
B	0.30757500	2.37592900	0.57194900
B	-2.58884000	-1.24549800	-0.50451100
B	2.44932900	-0.99000600	0.82098900
B	-1.61346900	-0.06007800	2.02416800
B	-1.31920400	-1.43538000	-1.62527700
B	-0.27605300	-1.16817600	2.00135000
B	2.63060400	-0.73408300	-1.04716800
B	-2.75169400	-0.27337300	0.84069200
B	3.55184200	0.00145100	0.09717400
B	-2.28066700	1.24907000	0.87579700
B	1.08951700	1.44189600	1.68826100
B	-1.32843000	2.40885200	0.33737400
B	-1.47671300	-2.40725500	-0.31189900
B	-0.36705600	2.40899100	-0.97129800
B	2.53668800	0.66353500	1.19315600
B	1.17145200	-0.25498900	1.73599000
Ti	-0.39272400	0.03431500	0.01569300

(2c) Ti@B_{28} (C_1, ^1A)

B	-0.88253900	-1.37678900	-1.61640200
B	1.90413500	-2.21930100	-0.84299200
B	1.28273000	2.38755800	0.71379400
B	-1.59418600	-0.25019100	2.78353200
B	0.42098700	1.49137900	-1.90797200
B	1.37485800	-0.91026400	1.77742700
B	2.71364800	-0.16328000	0.91413100
B	2.89334600	-0.95825100	-0.77528300
B	-1.00699500	1.76198800	-0.73972200
B	-0.09163800	-0.35438900	2.24919300
B	-2.23523800	-0.51492400	-2.10202600
B	2.49647700	1.42590100	0.70963400
B	-1.10679700	1.07053800	1.83690500
B	-1.57202100	-0.85134600	1.21214100
B	1.81162600	1.80331200	-0.99690400
B	-3.48016800	-0.32635800	-1.10333800
B	-3.27325800	0.09122300	0.40999200
B	-0.78359200	0.42120800	-2.00227200
B	-2.14936600	-1.11956100	-0.36828300
B	0.79671600	-2.22730900	0.52274300
B	0.35622300	2.56553900	-0.59671900
B	-2.72647300	0.34799300	1.89657600
B	-2.29268100	0.85797300	-1.10733000
B	2.29556200	-1.71446400	0.69887800
B	-0.80117200	-1.90312200	0.16916500
B	2.93208000	0.59331100	-0.74366900
B	-0.33126400	2.08004300	0.87507700
B	0.40527500	-2.19581300	-1.28422400
Ti	0.60084600	0.04259000	-0.13228500

(2d) Ti@B_{28} (C_1, ^1A)

B	-0.49961400	0.01524400	-2.34598000
B	2.18603300	0.41573000	-1.22039300
B	-0.46143600	-2.49618100	-0.41241100
B	-0.46301800	1.65957100	-1.94468000
B	0.01790500	1.55239500	1.80280900
B	0.96474000	0.94937600	-2.15435000
B	3.48166700	-0.16840700	-0.31481300
B	-1.52764800	-0.84090600	2.09754500
B	-2.74731500	-0.23827800	-0.76687500
B	-0.14136100	-1.71691700	1.96423700
B	1.04613800	-0.66784600	-1.86086400
B	1.29876800	-1.07917900	1.95901200
B	1.42002300	0.56974500	1.70767500
B	-1.89417900	0.90003500	-1.56328100
B	-0.07599400	-0.01554700	2.38658900
B	-1.42621900	2.00637800	1.03894300
B	-0.00539000	2.55551700	0.38367700
B	-1.25632400	2.20106100	-0.64111900
B	1.45596200	2.05274900	0.82140200
B	-1.33302500	-2.07900100	0.90219500
B	-1.90259500	-1.62747900	-0.62466100
B	-0.47246400	-1.51999700	-1.79535700
B	2.73043600	1.06108600	0.42887600
B	-2.40359500	1.08138200	0.20197900
B	2.42002700	-1.29002400	-0.81317800
B	-2.44431400	-0.64197200	0.80777600
B	2.39003700	-0.56305100	0.89604200
B	0.99547800	-2.14966400	-1.07878700
Ti	-0.30743700	0.01685900	0.03136200

(2e) Ti@B_{28} (C_1, ^1A)

B	1.33915600	-2.02359600	0.33465100
B	-1.17389500	1.66428900	0.98469200
B	1.02259100	2.55941800	-0.68711800
B	-0.57771500	2.44071100	-0.49179600
B	0.53897500	-0.85586900	-2.38318600
B	-0.04189700	1.28651900	2.14945300
B	1.57874200	1.03846600	1.71372700
B	2.67520400	-1.10452600	0.28912800
B	1.80722400	0.11807500	-2.18651400
B	-3.77629300	-0.24066800	-0.05868900
B	-1.37309300	-1.64193600	-0.41733600
B	2.10036600	1.61441000	0.26549100
B	-2.06492400	1.89743100	-0.72590500
B	-2.45724300	0.69106800	0.52668000
B	2.00765900	1.53466700	-1.44062500
B	0.20071500	-1.98993300	-1.12897600
B	-0.99388200	-0.93051400	-1.90194800
B	1.81734200	-1.25797400	-1.21714800
B	-3.37127000	1.00558200	-0.91401000
B	0.23277800	-1.61690800	1.77756000
B	-2.32769300	-0.40369000	-1.12713900
B	-2.74810100	-1.04888100	0.80362500
B	-0.21181800	-2.50555200	0.42398800
B	0.59725700	-0.14869400	2.46347100
B	-1.36077100	-1.69351100	1.31572700
B	2.69425100	0.16707600	-0.72741400
B	0.46838700	2.18811500	0.84387700
B	1.81149100	-0.82274400	1.63580700
Ti	0.36055800	0.01799300	-0.02728900

(3a) V@B_{28}^{+} (C_2, ^{1}A)

B	-0.51297100	-0.84165700	-2.24172500
B	1.59562900	1.54783300	0.46045400
B	1.83661600	0.76233900	2.03532000
B	0.00000000	2.33234100	0.41047400
B	-0.80078200	-1.96754200	1.92287100
B	0.69213300	1.89529400	-0.98001500
B	1.73513800	0.59802200	-0.97438500
B	1.57711500	-0.69693800	-2.32518100
B	0.79844900	-2.08679200	1.89528000
B	-0.79844900	2.08679200	1.89528000
B	-1.73513800	-0.59802200	-0.97438500
B	2.27089100	-0.07212300	0.53969000
B	-1.79436700	0.82802900	2.01761300
B	-1.69434600	1.71588300	0.51408900
B	1.69434600	-1.71588300	0.51408900
B	-1.59562900	-1.54783300	0.46045400
B	-1.83661600	-0.76233900	2.03532000
B	0.00000000	-2.33234100	0.41047400
B	-2.27089100	0.07212300	0.53969000
B	-0.67084100	0.39017800	-3.53827800
B	1.79436700	-0.82802900	2.01761300
B	-2.20043800	1.04008000	-0.86598600
B	-0.69213300	-1.89529400	-0.98001500
B	0.51297100	0.84165700	-2.24172500
B	-1.57711500	0.69693800	-2.32518100
B	2.20043800	-1.04008000	-0.86598600
B	0.80078200	1.96754200	1.92287100
B	0.67084100	-0.39017800	-3.53827800
V	0.00000000	0.00000000	0.49120800

(3b) V@B_{28}^{+} (C_1, ^{1}A)

B	1.21776900	0.10994500	-1.64173400
B	-0.14883000	0.60615300	2.34818200
B	1.01541500	-1.79139400	0.86459700
B	-0.15102000	1.03410900	-2.04334100
B	0.10202100	-2.32347000	-0.57708200
B	1.16277500	1.71698400	-0.95598400
B	1.22118500	-1.58865500	-1.54158800
B	-0.48101000	-2.36164700	1.03397200
B	2.53871900	0.85399500	-0.78100100
B	-2.60282100	0.30199300	-0.76108300
B	-1.67306800	1.51182000	-1.30358500
B	-0.13159900	-0.63973800	-2.24753100
B	0.33283500	2.40716100	0.48059700
B	-2.62710300	-1.27241600	-0.39930700
B	2.45359900	-0.98669500	0.85501100
B	-1.60712300	0.05126800	2.00837100
B	-1.40008300	-1.40344500	-1.53625800
B	-0.28143000	-1.06097500	2.02218000
B	2.63416700	-0.82630200	-1.01719000
B	-2.75774500	-0.20154500	0.85458600
B	3.56458200	-0.06303200	0.09807000
B	-2.23948800	1.30011300	0.81040300
B	1.13445300	1.51771900	1.61039000
B	-1.29981500	2.43720000	0.22508700
B	-1.50306200	-2.41321800	-0.21878500
B	-0.32057900	2.36466100	-1.06558600
B	2.56784800	0.68602900	1.15068000
B	1.15180200	-0.17885100	1.68199700
V	-0.40704200	0.04613800	0.00998500

(3c) $V@B_{28}^{+}$ (C_1, ^1A)

B	1.22398200	-1.99277000	-0.70535500
B	-3.35278200	-0.49330100	0.33255800
B	1.43675200	1.93237400	0.68715900
B	-0.43840500	-2.04797400	-0.69301800
B	-1.94066200	-1.35715900	-0.30829900
B	-1.78056600	1.29216300	0.30927300
B	-0.26800400	2.04348900	0.64979200
B	2.64830900	0.70553100	0.26628100
B	2.55221100	-0.90217600	-0.33280500
B	1.89717900	-1.94092900	0.88259100
B	-2.70469300	-1.66033000	1.20165700
B	0.55371300	1.46974300	2.05204700
B	0.38470500	-2.43365100	0.77091100
B	-1.16455400	-2.11586600	1.08885700
B	-1.89000300	-0.25118400	1.51161500
B	-0.95092300	0.96230900	1.79917700
B	1.73854300	0.46984200	2.03000100
B	2.65221000	-0.59874400	1.35297200
B	1.66217600	-0.60015200	-2.10330700
B	0.60837900	2.43862300	-0.78480100
B	0.34421700	-1.40898300	-2.07918100
B	-1.13715800	-0.88246300	-1.76857400
B	-0.93730300	2.23334700	-1.03725200
B	2.09726300	1.84882700	-0.87145500
B	2.61643500	0.41606700	-1.41776500
B	-2.49791200	1.82364200	-1.18118800
B	-3.46017700	0.96842600	-0.33654600
B	-2.31492200	0.06781200	-1.28090700
V	0.52652000	0.00293200	-0.00748700

(3d) $V@B_{28}^{+}$ (C_1, ^1A)

B	-1.60631100	-0.57152800	-1.94708200
B	1.13695000	-0.74160900	1.60804100
B	2.53480000	0.20201900	1.15379800
B	-0.26631600	-0.04062300	2.34405500
B	1.62799600	1.95779900	-0.73632800
B	-0.30377100	-1.61109000	1.62049300
B	-1.68738500	-0.70441100	1.90009700
B	0.24905800	-2.34830500	-0.84869700
B	1.27046800	0.43523100	-1.61524000
B	-1.21063900	2.30355800	0.94509400
B	-0.11882600	0.00069000	-2.31296400
B	0.97289300	-2.18930700	0.66315900
B	-1.29513100	1.99093200	-0.74945200
B	-0.01585600	1.52676800	1.80675400
B	3.62223600	-0.36395200	0.06950100
B	1.04494700	-1.17234700	-1.69950000
B	0.22825900	2.20916800	0.18122900
B	2.47570600	-0.65685300	-1.00202900
B	-2.43431200	0.59814300	-0.95669800
B	-2.57572500	-1.06622700	-0.76390700
B	2.90121200	1.00611800	-0.47817500
B	-2.44269900	1.47878400	0.41535800
B	0.08614400	1.62764400	-1.61436900
B	-1.89723000	-1.70438200	0.63408400
B	-2.66997600	-0.14198800	0.65991000
B	2.49067500	-1.39606600	0.66494000
B	1.45717100	1.42736200	0.97646300
B	-1.35585800	-2.07383500	-0.95862000
V	-0.48227800	0.00398000	0.00871500

(3e) V@B_{28}^{+} (C_1, ^1A)

B	-1.78160600	1.31873100	-0.89633800
B	1.43023200	1.91552300	-0.88941400
B	2.76660800	-0.48040500	-0.11179500
B	1.88900700	-1.15357000	-1.64048900
B	1.74368900	-0.31402000	2.21948200
B	0.54681900	-0.39863500	-2.09719100
B	-0.87921700	0.33144000	-1.86917600
B	-2.75379200	2.08698600	0.36361000
B	2.61327500	0.63026500	1.34135900
B	2.00735000	-1.90889200	-0.15473500
B	-0.77708700	-1.72155900	1.18971700
B	-0.19228200	1.75616600	-1.11813800
B	0.67447100	-2.26794300	0.72481000
B	0.60139300	-1.97118500	-1.19796100
B	-1.26525800	2.49424000	0.35513300
B	0.43384100	-1.13571400	2.13863300
B	2.71980600	1.13181600	-0.20919200
B	-1.75794300	0.92293900	1.24236700
B	-0.72636100	-2.45456100	-0.39149500
B	-3.46198700	-1.01166000	-0.26158400
B	1.76748000	1.98264800	0.86363300
B	-2.30601700	-2.00767200	-0.49077100
B	-0.95734600	-0.22747600	1.96494700
B	-2.20102500	-0.32657600	-1.16612300
B	-2.14616800	-0.72668900	0.83212800
B	0.38144600	2.53003200	0.36624500
B	2.02876500	0.49247400	-1.55575500
B	-3.22442200	0.56799300	0.06975500
V	0.61441900	-0.01189100	0.08224800

(4a) V@B_{29}^{2-} (C_s, $^1A'$)

B	-0.24585400	2.25690500	0.00000000
B	1.98951900	-0.95892800	1.72205400
B	2.71093800	0.15862400	-0.84329000
B	0.76519500	-1.83164400	-0.87480000
B	2.53444100	1.56144100	0.00000000
B	2.71093800	0.15862400	0.84329000
B	-1.42325900	1.26027200	0.82208400
B	1.98951900	-0.95892800	-1.72205400
B	-1.42325900	1.26027200	-0.82208400
B	-0.85025100	-1.45817100	-1.54070900
B	0.60456200	-1.11945300	-2.52620100
B	-0.89618000	0.44826200	2.21728300
B	-2.73983600	0.16780100	-1.41346800
B	-2.17690200	-0.74134500	-2.59107400
B	-2.73983600	0.16780100	1.41346800
B	-0.80750100	-0.99877000	-3.24958100
B	1.25332700	2.16644500	-0.85343900
B	-0.80750100	-0.99877000	3.24958100
B	-0.89618000	0.44826200	-2.21728300
B	-2.92591100	0.85939200	0.00000000
B	-0.05681000	1.77211900	1.71124100
B	-0.05681000	1.77211900	-1.71124100
B	0.60456200	-1.11945300	2.52620100
B	2.18735800	-1.25009500	0.00000000
B	1.25332700	2.16644500	0.85343900
B	-0.67358600	-1.94716000	0.00000000
B	0.76519500	-1.83164400	0.87480000
B	-0.85025100	-1.45817100	1.54070900
B	-2.17690200	-0.74134500	2.59107400
V	0.51694500	0.17154200	0.00000000

(4b) V@B_{29}^{2-} (C_1, ^1A)

B	0.05498300	0.21659000	2.24843600
B	-2.42783300	-0.92139300	-1.15748300
B	-0.58159900	-2.61485700	0.37733300
B	0.47221100	-1.14728600	-1.73441100
B	-1.61194900	-1.99885500	1.53068300
B	-2.14441500	-1.99083300	-0.00179500
B	0.00193900	1.67049800	1.26066300
B	0.55203500	-2.56776200	-0.76249000
B	1.39057100	0.78511400	1.27433500
B	1.85818600	-0.08616000	-1.40903200
B	1.97929500	-1.83164800	-0.98211000
B	-1.43842400	1.91837200	0.41251500
B	2.64387100	1.65559900	0.23716800
B	3.38323500	0.54577800	-0.61717300
B	0.18138500	3.03715200	0.13271200
B	3.30204000	-0.97686100	-0.88166700
B	-0.04767500	-1.57085900	1.97780800
B	-2.31970700	2.27594900	-1.10250000
B	2.34380600	-0.39755600	0.49259300
B	1.48869100	2.50667700	0.88257200
B	-1.47471900	0.99893800	1.78420700
B	1.38820800	-0.84012700	1.76721000
B	-2.39715800	0.68059000	-1.25111000
B	-0.99998700	-1.93855200	-1.23813400
B	-1.45147300	-0.55481500	2.24421600
B	0.44792100	0.53095900	-1.94134100
B	-1.03218300	-0.27533300	-1.92892500
B	-0.81082400	1.46243300	-1.50477200
B	-1.08692100	3.15542100	-0.83241500
V	-0.36163200	-0.37547300	0.15715300

(4c) V@B_{29}^{2-} (C_1, ^1A)

B	-0.88087100	-0.37489900	1.56364200
B	2.48505300	1.24231800	0.83080500
B	-1.81714300	2.17147600	0.82638700
B	1.14667100	1.23051800	-1.88151000
B	0.05321600	-1.82474400	1.71608900
B	-1.33217600	-1.95463700	0.90173700
B	-0.46220000	1.31599400	1.52980800
B	-1.81714000	2.17132800	-0.82682900
B	-2.55172500	-0.65883500	0.94583500
B	-2.72408500	-2.05394700	0.00017800
B	-2.55169700	-0.65902400	-0.94575100
B	0.05327500	-1.82507800	-1.71569400
B	1.32920200	2.14082800	-0.00002100
B	-0.46219300	1.31572200	-1.53008500
B	-2.20923700	0.78562300	-1.68329000
B	-0.30133800	2.20244900	-0.00021900
B	1.14669700	1.23092900	1.88115600
B	2.28522400	-0.06472100	1.73785500
B	-2.38861100	0.80828200	-0.00009800
B	0.79163300	-0.48019700	2.28991500
B	-2.20927200	0.78595000	1.68310700
B	-0.88082700	-0.37520000	-1.56357100
B	2.28523700	-0.06501400	-1.73780100
B	0.79168800	-0.48069000	-2.28985200
B	1.74190400	-1.64875300	1.49943700
B	-1.33213600	-1.95481100	-0.90134700
B	2.03255300	-1.99235800	0.00021700
B	1.74198600	-1.64901700	-1.49907400
B	2.48494500	1.24229200	-0.83109300
V	0.77203600	-0.12647500	0.00001400

(4d) V@B_{29}^{2-} (C_1, ^{1}A)

B	-0.02844700	0.06184300	2.20048400
B	-0.20685600	-2.39853100	-1.02823700
B	2.41581500	-1.75843700	-0.13212700
B	0.55665400	-0.91208300	-2.07221100
B	1.87710400	-1.95505000	1.37277400
B	0.87468500	-2.61052100	0.18890800
B	-1.39755400	0.49293500	1.30327900
B	2.27815700	-0.66288300	-1.35127800
B	-0.10379300	1.51894600	1.27532800
B	-0.99243000	1.32160700	-0.64119600
B	2.20646800	0.92868200	-1.30152000
B	-2.40382500	-0.73919900	0.58501100
B	-0.31628000	2.93903500	0.16553100
B	1.01341400	3.40773500	-0.56158600
B	-2.75194800	1.13556900	0.22864700
B	2.14828400	2.49857600	-1.07215600
B	1.53844200	-0.57059000	2.13842100
B	-3.16822800	-1.32789500	-0.97764100
B	1.36711900	2.11461700	0.57306500
B	-1.66392500	2.23390700	0.72843200
B	-1.24106200	-1.16364500	1.68842700
B	1.47071200	0.97405800	1.73508800
B	-1.71141500	-1.88373500	-1.06313400
B	1.37340900	-2.06576900	-1.36809800
B	0.25674100	-1.74557900	1.79705800
B	0.57938400	1.73496300	-1.24916700
B	-0.38998900	0.40336300	-1.89492700
B	-1.94330800	-0.05367300	-1.15636600
B	-3.64712200	0.03479500	-0.43523400
V	0.43691200	-0.42457400	0.07052700

(4e) V@B_{29}^{2-} (C_1, ^1A)

B	-1.66175700	1.32323700	1.28536500
B	0.26580300	0.30263500	-2.14044000
B	-1.84279900	-1.28544700	-0.91545100
B	0.89341600	-1.29292600	-1.62106800
B	0.57977500	2.68451600	-0.23017500
B	2.75836600	1.06808100	0.88134300
B	-2.60856500	2.43841000	0.46575700
B	-1.40086600	-2.94087700	-0.47324600
B	-1.64715300	-0.41540900	0.87720000
B	-3.25381000	-0.80269300	0.17407100
B	-1.26825800	0.14951700	-1.51543200
B	2.04186700	2.12703600	-0.07042500
B	-0.31137900	-1.24959200	1.61279700
B	-0.37995100	-2.38090000	-1.47022200
B	0.09365900	-2.27280200	0.36213200
B	-2.71566100	-2.23048700	0.19351900
B	-0.41318400	1.65052700	-1.27615200
B	1.66173100	-1.91729500	-0.12964600
B	-3.05120400	0.83367300	0.34215400
B	-0.65354000	0.41698900	2.11836000
B	-1.08751000	2.76048700	0.03406900
B	-1.98055800	1.52810300	-0.80212200
B	2.54902700	-0.89701200	-1.04642200
B	1.87642100	0.19508400	-2.00891300
B	1.23624100	1.64650200	-1.54491200
B	1.40104000	-1.40994700	1.47602000
B	2.71873600	-0.73950900	0.56440100
B	2.28551600	0.10365300	1.99551500
B	0.75689300	-0.19901400	2.45443700
V	0.68645800	0.17510000	0.08858400

Cs V@B_{29}^{-}

B	-0.23630200	2.23844500	0.00000000
B	2.00237500	-0.95154200	1.70494900
B	2.65073200	0.22813900	-0.85581000
B	0.78921800	-1.83976100	-0.87267400
B	2.54460600	1.62263000	0.00000000
B	2.65073200	0.22813900	0.85581000
B	-1.48091100	1.34370700	0.85097800
B	2.00237500	-0.95154200	-1.70494900
B	-1.48091100	1.34370700	-0.85097800
B	-0.81808400	-1.48444400	-1.53459200
B	0.62991500	-1.16026500	-2.54227000
B	-0.89667400	0.41240100	2.15501800
B	-2.69941500	0.14367400	-1.38360800
B	-2.16002000	-0.78686800	-2.55126200
B	-2.69941500	0.14367400	1.38360800
B	-0.79212300	-0.99023900	-3.22971500
B	1.25596400	2.15629500	-0.86410100
B	-0.79212300	-0.99023900	3.22971500
B	-0.89667400	0.41240100	-2.15501800
B	-2.90879100	0.90970400	0.00000000
B	-0.07225700	1.77066200	1.68820400
B	-0.07225700	1.77066200	-1.68820400
B	0.62991500	-1.16026500	2.54227000
B	2.19464000	-1.22399700	0.00000000
B	1.25596400	2.15629500	0.86410100
B	-0.64251600	-1.98098400	0.00000000
B	0.78921800	-1.83976100	0.87267400
B	-0.81808400	-1.48444400	1.53459200
B	-2.16002000	-0.78686800	2.55126200
V	0.48498300	0.16319200	0.00000000

C_1 Ti@B_{28}^{-}

B	2.18701200	-0.48149900	0.90559900
B	-0.39038400	1.21758200	-1.91903400
B	-1.89754900	1.77714200	-1.17200500
B	-0.50309700	-0.53821300	-2.26040900
B	-1.88714400	-0.14052200	2.12140200
B	0.95475700	0.16547900	-2.04559600
B	1.06213100	1.45035100	-0.98642100
B	2.43915300	1.66973700	0.17778000
B	-1.70907400	1.42939200	1.83028000
B	-2.02282000	-1.12978500	-1.73606100
B	0.86516000	-1.65183300	1.00211200
B	-0.34435100	2.31480500	-0.50585900
B	-2.15394100	-1.85028400	-0.29687300
B	-0.69682700	-2.06144300	-1.22873700
B	-0.23586400	2.06912800	1.20508500
B	-0.50906000	-1.16924200	1.94684700
B	-2.10287700	-1.46834900	1.26043600
B	-0.29125300	0.60976100	2.24796200
B	-0.69819700	-2.25886600	0.52315500
B	3.44353900	-0.97167700	-0.24029600
B	-1.80631600	2.11609800	0.38443400
B	0.67068400	-2.50356400	-0.46139000
B	0.99509600	-0.32945100	2.01957600
B	2.16822800	0.10591400	-0.92130100
B	2.16002500	-1.86294700	-0.29854400
B	1.12234600	2.60371100	0.32689700
B	-1.93046100	0.44473900	-2.06424200
B	3.51083400	0.51990800	0.17707800
Ti	-0.54539800	-0.01728900	0.00184600
